# A Pilot Study on Secondhand Smoke Exposure Among Pregnant Women in Chongqing, China: A Combined Questionnaire, Saliva Cotinine Test, and Ultrasound Flow Index Analysis

**DOI:** 10.3389/fpubh.2020.00290

**Published:** 2020-07-29

**Authors:** Jing Tang, Jie Shen, Shengjie Zhang, Harvey Ho, Suzhen Ran

**Affiliations:** ^1^Chongqing Health Center for Women and Children, Chongqing, China; ^2^Auckland Bioengineering Institute, The University of Auckland, Auckland, New Zealand

**Keywords:** secondhand smoke, pregnant women, nicotine, cotinine, ultrasound, umbilical artery

## Abstract

**Objective:** The aim of the study was to gain updated data on SHS exposure among pregnant women in Chongqing city, one of the four municipalities of China.

**Study Design and Setting:** Pregnant women attending routine ultrasound checks at an obstetrics and gynecology center voluntarily participated in the survey of SHS exposure. Some participants were also invited to have saliva cotinine tests using a NicAlert kit. The pulsatility index (PI) of the umbilical artery was compared between the SHS and non-SHS groups.

**Results:** A total of 548 pregnant women (74.82 and 25.18% at 12 and 24 weeks' gestation, respectively) participated in the survey from July to November 2019. SHS exposure was reported by 29.44% of participants. “Workplace” was listed as the top location for SHS exposure. Twenty-three of the 31 participants who tested cotinine-negative in their saliva samples self-reported SHS exposure in the survey questionnaire. However, two of the eight participants who tested cotinine-positive in their saliva samples self-reported SHS-exposure negative. The mean PI in the SHS group is higher than that in the non-SHS group (1.10 vs. 1.02) in fetuses at 24 weeks' gestation. However, the PI may not be suitable as an index for SHS exposure at 12 weeks' gestation due to frequent absent or reversed diastolic flow.

**Conclusion:** The first survey on SHS exposure in pregnant women that combines a questionnaire, saliva cotinine tests, and ultrasound flow index analysis has been performed in China and provided valuable data for ensuing studies.

## What Is New?

- The first survey on SHS exposure in pregnant women that combines a questionnaire, objective saliva cotinine tests, and ultrasound flow index analysis has been performed in China;
- 29.44% of 548 participants reported SHS exposure of more than 15 min per day;
- “Workplace” (42.15%) was the top venue where SHS exposure occurred;
- The prevalence of 31 women detected SHS-exposure positive by saliva cotinine test was 25.8%;
- Six (75%) participants whose saliva samples were detected SHS-exposure positive reported SHS exposure;
- The mean PI in the SHS group is higher than that in the non-SHS group (1.10 vs. 1.02) in fetuses at 24 weeks' gestation.

## Introduction

China is the world's largest producer and consumer of tobacco, yet the prevalence of active smoking among Chinese women is about 3.84% (1), much lower than the ~10% in the majority of developed countries (2). Rather, Chinese women, including pregnant women, are more likely to be exposed to secondhand smoke (SHS): according to the Global Adult Tobacco Survey in China in 2010, 65.1% of non-smoking women of childbearing age were exposed to SHS at home, and another 52.6% were exposed to SHS in the workplace (3). The prevalence of SHS exposure among pregnant women ranged from 38.9 to 75.1% in eight studies performed between 2008 and 2012 (4).

Previous studies have shown the adverse effects of active or passive smoking on fetal health. For example, pregnant women exposed to smoking are more likely to have intrauterine growth restriction (IUGR) (2), placenta previa (5), and other perinatal diseases. A study in Taiwan for 278 pregnant women found that the mothers not exposed to smoking had children that weighed significantly more (3205.7 ± 373.1 g) than those of active smoker mothers (2959 ± 403.7 g) and those of SHS-exposed mothers (3089.7 ± 363.0 g) (6). Exposure to smoking is also associated with cardiovascular diseases of mothers and fetuses (7, 8). In a study of 60,377 women in Shanghai, the authors found that female non-smokers who lived with husbands who smoked had an elevated prevalence of stroke (8).

In view of the adverse effects of SHS exposure on fetal development, tobacco control policies and intervention guidelines need to be set forth in the communities and households of pregnant women. However, as pointed out by a recent survey of risk factors for cancers in 31 provinces of mainland China (9), in a country as large and populous as China, there are important differences in SHS exposure in different regions. Hence, it is essential to have region-specific, updated data on SHS exposure among pregnant women.

The primary aim of the study is to investigate the current SHS exposure situation among pregnant women in Chongqing, which is a metropolitan city in China with an urban population of 8.5 million and yet for which data for SHS exposure among pregnant women is lacking. Thus, we use a questionnaire-based approach to gain an overall picture of SHS exposure in this city. Since there are possible inaccuracies in self-reporting, we also experiment with a saliva cotinine kit for detecting SHS exposure among pregnant women—the first use of this kind of experiment in China. In addition, since flow indices of the umbilical artery (UA) are suggested to be a biomarker for pathological conditions of the placenta (10), we also investigate the flow indices revealed from Doppler sonography on the UA.

## Methods

### Study Design

The study was performed at the Chongqing Health Center for Women and Children (CHCWC), Chongqing City, China. The center manages ~16,000 childbirths each year. The Ultrasound Department of the CHCWC was in charge of the study, including conducting questionnaire management, saliva cotinine sample tests, and fetal ultrasonic scans. A team of biomedical scientists at the University of Auckland, New Zealand, assisted with the questionnaire design and statistical analysis. At the first stage, pregnant women at the nuchal translucency (NT) period (12 ± 1 weeks) and 24 ± 1 weeks of gestation could voluntarily participate in the study by signing a consent form and self-reporting their SHS status. A subset of this participant group also had their saliva cotinine measured and arterial flow indices of their fetuses analyzed. The study was approved by the Ethics Committee of the CHCWC.

### Questionnaire Design

A questionnaire on SHS exposure was designed and implemented in a mobile phone application, namely, Wen Juan Xing (“Questionnaire Star”) on WeChat, a Chinese social media platform. Prospective participants could scan a QR code of the application and enroll in the study by signing an electronic consent form. The questions on demographic parameters include age (in years), education level (above or below tertiary education), family income (under or above RMB 150k), and drug use history during pregnancy, if any. The questions concerning SHS exposure include:

1. Did you smoke before pregnant? If yes, approximately how many cigarettes did you smoke per day? (above or below 10 cigarettes).
2. Have you smoked after becoming pregnant? If yes, approximately how many cigarettes do you smoke per day? (above or below 10 cigarettes).
3. Does your husband smoke? If yes, approximately how many cigarettes does he smoke per day? (above or below 10 cigarettes).
4. If you are exposed to secondhand smoke, where does it happen (multiple choices allowed): (a) Home; (b) Workplace; (c) Restaurant; (d) Public transportation; (e).
5. If you are exposed to secondhand smoke, approximately how many minutes would it be? (a) Under 15 min (no SHS exposure); (b) 15–30 min; (c) 15–60 min; (d) above 60 min.

Answers to these questions as well as the demographic data were analyzed by SPSSAU, a software package that comes with Wen Juan Xing, which has similar statistical algorithms as are used in SPSS (IBM, Inc.).

### Saliva Cotinine Strip Tests

**Figure 1:**
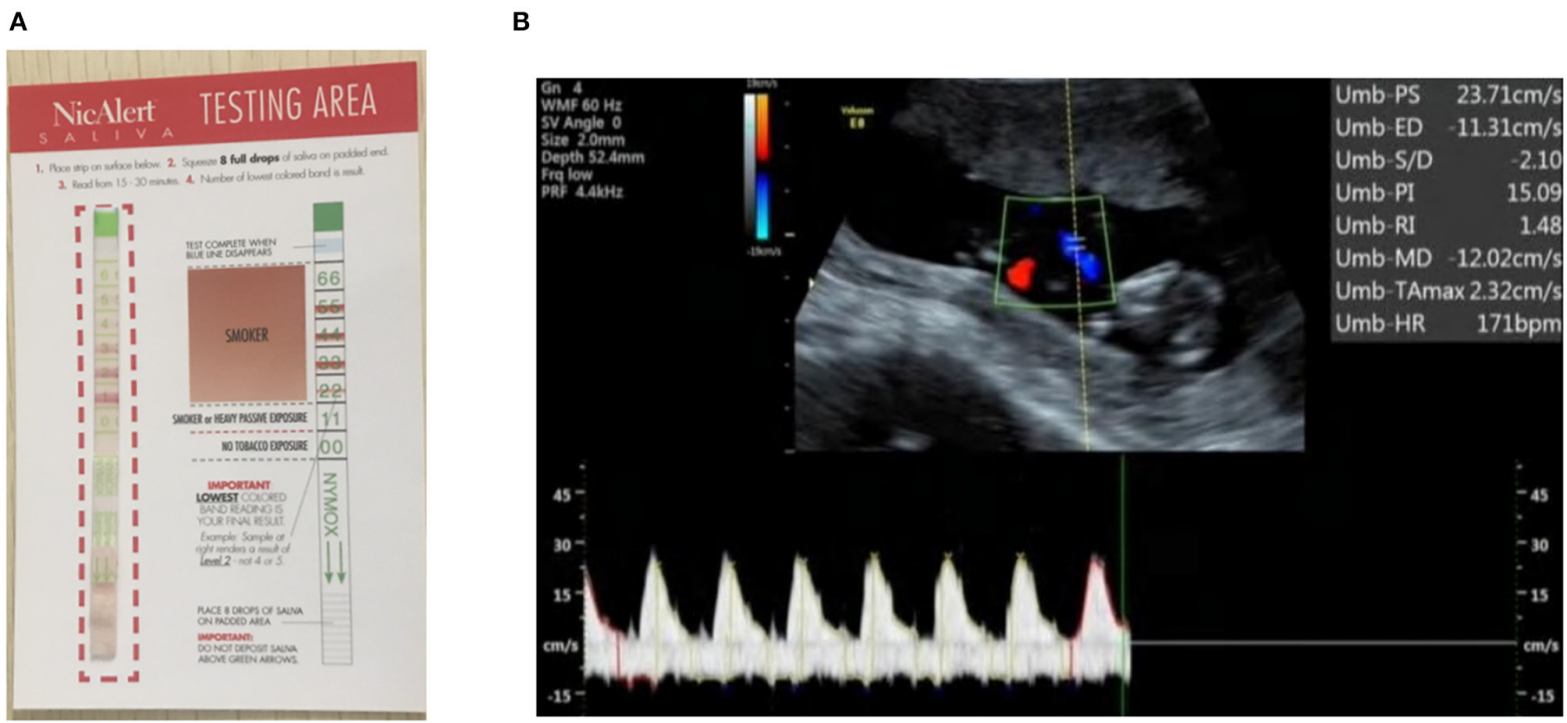
**(A)** A sample of a NicAlert saliva cotinine test strip: a reading of 1 or above (marked red on the strip) is grouped as positive SHS exposure in this study; **(B)** the flow indices for a fetal umbilical artery at the NT stage (12 weeks' gestation) are shown in the right upper corner. The end diastolic flow was negative, indicative of reversed flow, and resulted in abnormally high PI.

A saliva cotinine test kit (NicAlert^TM^, Confirm Bioscience, CA, USA) was used to quantitatively measure the cotinine level in saliva samples. As the major metabolite of nicotine, cotinine is often used as the biomarker of nicotine due to its much longer half-life (16 vs. 2 h) than nicotine (11). The test kit utilizes monoclonal antibody-coated gold particles and a series of avidity traps to determine cotinine concentration (12). With a cutoff cotinine detection concentration of 10 ng/ml, there are seven readings marked on the kit strip (from 0 to 6) (Figure 1A). In this study, we deem all readings above 1 as being positive for SHS exposure and 0 as negative exposure.

For the purpose of proof-of-concept, a small subset of the participants was selected for saliva cotinine tests. The selection criteria included: (a) no anatomic abnormality detected in the fetus from ultrasonography imaging; (b) the mother had no underlying diseases, e.g., diabetes and hypertension. Furthermore, the sample should include a group of participants who self-reported SHS exposure and a control group who did not report exposure to SHS. The sample size was restricted by the availability of saliva kits, of which there were 50 when the tests were performed.

### Ultrasound Scans

All survey participants underwent routine prenatal ultrasound scans in the Ultrasound Department of CHCWC; this was the main purpose of their hospital visits. From those participants who had their saliva samples measured, we retrospectively analyzed the flow indices revealed from Doppler sonography (GE Voluson^TM^ E8). Previous studies have shown that exposure to active smoking affects the systole/diastole flow velocity ratio (S/D ratio) in fetal umbilical and middle cerebral arteries (10, 13). However, the S/D ratio may not be available at the NT period due to the absent diastolic flow (the flow velocity at the end diastole is zero, thus rendering the S/D ratio invalid). We therefore use a pulsatility index [(peak systole velocity—end diastole velocity)/mean flow velocity] instead of the S/D ratio. However, when there are adverse flows at the diastole, the end diastole velocity becomes negative, which would yield an abnormally high PI.

## Results

### SHS Survey Results

During the 4 months from July to November 2019, 548 pregnant women participated in the survey. Of these, 74.82% were at the NT stage (12 ± 1 weeks' gestation), and the rest were at 24 ± 1 weeks' gestation. The average age of the participants was 29 years (SD 4.1). The active smoking rate before pregnancy was 3.47%, similar to the published active smoking rate of 3.84% among Chinese women (1). The rate dropped to 0.18% during pregnancy, suggesting the awareness of adverse effects of smoking on fetuses. Exposure of more than 15 min per day was reported by 29.44% (161/548) of participants, and 43% of the participants' husbands were smokers (Table 1). However, only 18.61% of all participants listed “Home” as the venue where SHS exposure may occur. Rather, the “Workplace” (42.15%) was the top venue where SHS exposure could happen, followed by “Public transportation” (33.94%) and “Restaurants” (26.64%). There was no statistically significant correlation between SHS exposure and education level (*P* = 0.465), and neither did we find a statistical correlation between SHS exposure and family income (*P* = 0.687).

**Table 1:** Demographic and SHS-related characteristics of participants (*n* = 548).

**Demographic characteristics**	
Age (Mean)	29 (4.1)
Education (Below Tertiary), *n* (%)	291 (53.1)
Annual income (< RMB150k)^*^, *n* (%)	358 (65.33)
Trimester 1 (0–12wk), *n* (%)	410 (74.82)
**Smoking-related characteristics**	
Smoking pre-pregnancy, *n* (%)	19 (3.47)
Smoking during pregnancy, *n* (%)	1 (0.18)
Partner being a smoker, *n* (%)	239 (43.61)
SHS exposure at home, *n* (%)	102 (18.61)
SHS exposure time (<15 min), *n* (%)	387 (70.62)
SHS exposure time (<30 min), *n* (%)	524 (95.62)

* *Income recorded in Chinese currency. 1 USD = 7 RMB*.

### Saliva Cotinine Test Results

Saliva cotinine levels were measured in 31 participants. All 23 (100%) pregnant women who tested SHS-exposure negative (i.e., saliva cotinine readings were zero) also self-reported the same. Among the eight participants whose saliva samples tested SHS-exposure positive (i.e., saliva cotinine readings were one or above), six (75%) reported SHS exposure of more than 15 min. However, two (25%) self-reported as negative for SHS exposure in the questionnaire.

### Flow Indices of UA

Among the pregnant women who had their saliva cotinine level measured, the flow indices of the fetuses of 23 of them were available for analysis. Eight of the 23 women had fetal ultrasound data at both 12 ± 1 and 24 ± 1 weeks' gestation. For this group, the PI at 24 ± 1 weeks had a mean of 1.03 (SD 0.12), lower than the PI at 12 ± 1 weeks' gestation, 3.39 (1.12). The abnormally high PI of the latter resulted from reversed diastolic flows (Figure 1B). The end diastolic flow velocity at the UA was zero in 10/21 fetuses and was negative in 11/21 fetuses at 12 ± 1 weeks' gestation. This phenomenon is normal for fetuses at the early stage of gestation. As gestation advances, the end diastolic flow increases. Indeed, zero or reversed flows did not occur in any of the 15 fetuses at 24 ± 1 weeks' gestation.

The comparison between the PI of the SHS-exposure positive and negative groups is listed in Table 2. At 24 ± 1 weeks' gestation, the PI is higher in the SHS group (mean 1.10, SD 0.27) than the non-SHS exposure group (mean 1.02, SD 0.11). However, the sample size of the SHS-exposure group was too small, and the results are not conclusive.

**Table 2:** The mean value and standard deviation of the flow indices of PI.

**Weeks of gestation**	**Flow indices**
**With SHS exposure (Total 7)**	
12 ± 1 (*n* = 5)	2.97 (0.85)
24 ± 1 (*n* = 2)	1.10 (0.27)
**Without SHS exposure (Total 29)**	
12 ± 1 (*n* = 16)	4.41 (3.23)
24 ± 1 (*n* = 13)	1.02 (0.11)

## Discussion and Conclusion

Exposure to active or passive smoking during pregnancy is associated with many detrimental effects on fetuses, including restricted fetal growth, stillbirth, preterm delivery, and sudden infant death syndrome, to name a few (8, 10). The aim of the study was to gain updated data on SHS exposure among pregnant women. Although Chongqing city is one of the four municipalities of China, no investigations of SHS exposure among pregnant women have been reported for this city, though one has been performed for another municipality city, Shanghai (8). The findings of the current study highlight the difficulties of pregnant Chinese women in avoiding exposure to SHS completely. While the Chinese central government and provincial administrations have been promoting smoking control policies over the past decade (14), the current study clearly shows that more of these still need to be implemented in public places. On the other hand, it is interesting to observe that while 43.61% of the pregnant women reported that their husbands were active smokers, only 18.61% of them listed their home environment as the venue for possible SHS exposure. This result is drastically different from another study performed a decade ago that reported that 65.1% of non-smoking women were exposed to SHS at home (3). This may suggest much-enhanced awareness of the potential harm of smoking to fetuses and that family members are refraining from smoking in the presence of pregnant women.

There are several novelties in the implementation of this study. Firstly, a mobile phone-based application was used to collect and analyze the data from the questionnaire, taking advantage of the ubiquity of mobile phones in present-day China. Unlike a paper-based questionnaire, a user could complete the survey at any time and any location at her convenience. Secondly, this work represents the first study using saliva cotinine strips for objective cotinine measurements among pregnant women in China. Saliva cotinine strips have been used in studies in New Zealand (12) and Switzerland (15) for smokers/non-smokers but not specifically for pregnant women. A study in the United States made use of urine cotinine strips in comparison with gas chromatography, the gold standard for determining cotinine levels in urine (16). However, saliva sample preparation is more convenient than that of urine samples and takes a shorter time.

Previous studies suggested that up to 35% of pregnant women might self-report inaccurately due to embarrassment or shame (17); thus, it is important to verify self-reported questionnaire results. While the use of cotinine strips or dipsticks has the advantages of non-invasiveness, low cost, and needing minimal training and equipment (12, 16), it may overestimate the cotinine concentration, as pointed out by a recent study on the high false-positive rate in detecting cotinine levels of smoking with NicAlert (15). Hence, we were not able to determine the actual SHS exposure status for the two pregnant women whose saliva samples tested SHS exposure-positive but self-reported negative. Gas or liquid chromatography is required to further check the results, which was not within the scope of the original study design but could be implemented in future studies.

Thirdly, the analysis for flow indices in fetuses started at the NT period, i.e., end of the first trimester, whereas most of the literature reports flow index data from middle to late gestation, e.g., 34–35 weeks in (10), 23–40 weeks in (13). Due to the limited number (23) of fetuses, the analysis here in particular for the SHS-exposure positive group was not conclusive, and more fetuses should be included in further study. However, both the S/D ratio and PI in UAs may not be suitable for SHS-exposure related analysis at the NT period due to zero or negative end diastolic flows. In general, the end diastolic flow velocity increases as gestation advances, and the flow velocity waveforms become less pulsatile; thus the PI and S/D ratio tend to decrease as gestation continues (18).

There are limitations pertaining to the current study. Firstly, while 548 pregnant women took part in the SHS survey, only a small portion of the participants received saliva cotinine tests, and only 8 of the 23 women had fetal ultrasound data at both 12 ± 1 and 24 ± 1 weeks' gestation. A larger sample size is required to verify the findings of this preliminary investigation. Secondly, pregnant women in their first and second semesters were selected in this pilot study because of the timeframe (Jul–Nov 2019), and we could longitudinally trace the pregnant women in the third trimester in future studies. In addition, some essential data, e.g., the efficacy of the saliva cotinine kit, were required that would help us to design the questionnaires of the ensuing series. Interestingly, ultrasound data for UAs from the literature were mainly collected from pregnant women in the late second and the third semester (10, 13). Therefore, the data collected herein in the first half of pregnancy could fill this data gap.

In conclusion, the results and data from this pilot study are not only valuable for guiding our ensuing studies on SHS exposure but may also be useful as a reference for public health workers for the implementation of SHS intervention in public places.

## Data Availability Statement

The datasets generated for this study are available on request to the corresponding author.

## Ethics Statement

The studies involving human participants were reviewed and approved by The Ethics Committee of the Chongqing Health Center for Women and Children. The patients/participants provided their written informed consent to participate in this study.

## Author Contributions

JT: Survey questionnaire design, fetal ultrasound scan, and analysis. JS: Data collection, saliva cotinine tests, and fetal ultrasound scan. SR: Fetal ultrasound scan and project management. HH: Survey questionnaire design, ultrasound data analysis, and paper drafting. SZ: Statistical analysis and paper drafting. All authors: reviewed and agreed the paper.

## Conflict of Interest

The authors declare that the research was conducted in the absence of any commercial or financial relationships that could be construed as a potential conflict of interest.
